# In Response to a Punctual Stress Male and Female Tyrosine Hydroxylase Haploinsufficient Mice Show a Deteriorated Behavior, Immunity, and Redox State

**DOI:** 10.3390/ijms24087335

**Published:** 2023-04-15

**Authors:** Judith Félix, Antonio Garrido, Mónica De la Fuente

**Affiliations:** 1 Animal Physiology Unit, Department of Genetics, Physiology and Microbiology, Faculty of Biological Sciences, Complutense University of Madrid, 28040 Madrid, Spain; jufelix@ucm.es (J.F.);; 2 Institute of Investigation Hospital 12 Octubre (imas12), 28041 Madrid, Spain; 3 Department of Health Sciences, Faculty of Biomedical and Health Sciences, Universidad Europea de Madrid, 28670 Madrid, Spain

**Keywords:** punctual stress, tyrosine hydroxylase haploinsufficiency, behavior, immune functions, oxidative stress, sex, restrain

## Abstract

An inadequate stress response is associated with impaired neuroimmunoendocrine communication, increasing morbidity and mortality. Since catecholamines (CA) constitute one of the acute stress response pathways, female mice with an haploinsufficiency of the tyrosine hydroxylase gene (TH-HZ), the main limiting enzyme in CA synthesis, show low CA amounts, exhibiting an impairment of homeostatic systems. The aim of this study was to investigate the effect of a punctual stress in TH-HZ mice, determining the differences with wild-type (WT) mice and those due to sex by restraint with a clamp for 10 min. After restraint, a behavioral battery was performed, and several immune functions, redox state parameters, and CA amounts were evaluated in peritoneal leukocytes. Results show that this punctual stress impaired WT behavior and improved female WT immunity and oxidative stress, whereas in TH-HZ mice, all parameters were impaired. In addition, different responses to stress due to sex were observed, with males having a worse response. In conclusion, this study confirms that a correct CA synthesis is necessary to deal with stress, and that when a positive stress (eustress) occurs, individuals may improve their immune function and oxidative state. Furthermore, it shows that the response to the same stressor is different according to sex.

## 1. Introduction

The stress response is defined as a reaction to a perceived threat to homeostasis [1]. However, it is commonly associated with negative effects that threaten health without considering that a stressful situation can also be positive for adaptation to the environment and for anticipating the different challenges of life [2,3]. Accordingly, a good way to classify stress is into eustress (good stress) and distress (bad stress), so that distress would involve a situation that could impair normal physiological functions and even lead to pathological conditions, whereas eustress could benefit health through the optimization of homeostasis induced by hormesis. Consequently, an optimal level of stress is essential for building biological shields to ensure normal vital processes [2]. Now, considering that the maintenance of health depends on the correct performance of the homeostatic systems (nervous, immune, and endocrine) and the correct communication between them [4,5], and that the stress response is directly associated with homeostasis, it can be asserted that a deficient response to stress would produce an alteration in the neuroimmunoendocrine communication, which would increase morbidity and mortality [4,6]. Biologically, the stress response is mediated by different mechanisms that lead to the activation of the hypothalamic–pituitary–adrenal (HPA) axis and the sympathetic–adreno–medullary (SAM) axis. This work will be focused only on the SAM axis since it is responsible for the production of catecholamines (CA), constituting one of the main response pathways to acute stress within neuroimmunoendocrine communication thanks to the receptors that the organism presents both centrally and peripherally [7,8,9]. In the nervous system, CAs, mainly noradrenaline (NA) and dopamine (DA), are responsible for the regulation of mood, motivation, arousal, and plasticity, as well as for the development of various functions such as cognition, attention, the anxiety response, memory formation, and locomotion control [10,11]. In the immune system, CAs act mainly by regulating both innate and adaptive immunity [12,13]. 

The catecholamine pathway can be limited by several factors, but one of them can be an inactivation of tyrosine hydroxylase (TH), the first enzyme in the synthesis of CA [14]. In this background, it has been observed that mice that are hemizygotes for the gene that synthesizes CA (TH), the TH-HZ mice, show a decrease in TH activity, resulting in a lower production of CA, which has been associated with an impairment of the homeostatic systems. This is associated with impaired sensorimotor skills, decreased exploration, and increased anxiety levels accompanied by the establishment of premature immunosenescence and oxidative-inflammatory stress leading to accelerated aging and, therefore, a lower longevity compared to their corresponding wild type (WT) counterparts [15,16,17].

To study the different responses that various stressors can have on the interactions that occur between the nervous, immune, and endocrine systems, different animal models have been proposed. Some of the most commonly employed stressors are restraint, social disruption, cold exposure, restraint with cold exposure, electric shock, forced swimming, food deprivation, wet sawdust, cage agitation, cat odor exposure, or reversal of the light/dark cycle [18,19,20,21,22,23]. Most of these methods report different effects that acute stress can have on animals, such that if they involve eustress for them, they will benefit the health of the animals, whereas if they involve distress, they could impair it. However, these protocols have not been applied to animal models that present an altered stress pathway, such as TH-HZ mice, so it is not possible to know whether individuals with this genetic alteration would have a similar response to a stressor as WT individuals. Based on that, the aim of the present study was to investigate the effect in the behavior, immunity, and oxidative state of an acute stress by restraint in TH-HZ mice, and to determine the differences compared to the response in WT mice. In addition, it is known that there are sex differences in the functions of homeostatic systems and in the involvement of these systems in the response to stress situations [24]. Since these sex differences in the stress response of TH-HZ mice are still unknown, this aspect was also studied. 

## 2. Results

### 2.1. Behavioral Tests

**Figure 1:**
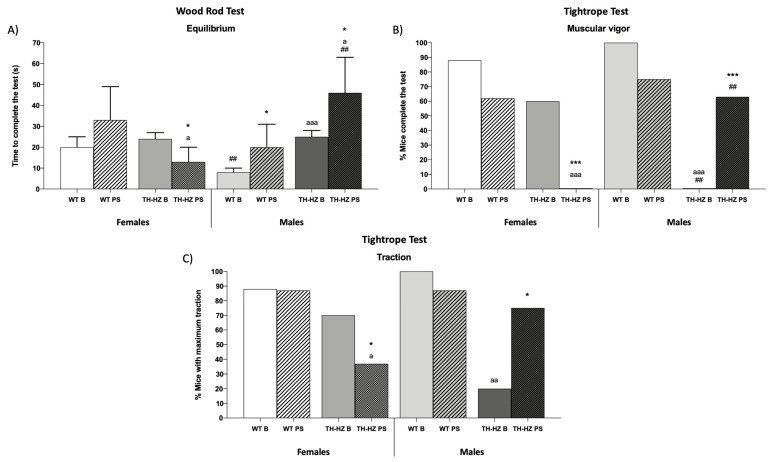
Sensorimotor abilities. (**A**) Time (in seconds) to complete the wood rod test. (**B**) Percentage (%) of animals that completed the tightrope test. (**C**) Percentage (%) animals that performed the tightrope test with the maximum traction. Each column represents the mean ± standard deviation of values corresponding to 6 animals. * *p* < 0.05, *** *p* < 0.001 compared to basal condition, a *p* < 0.05, aa *p* < 0.01, aaa *p* < 0.001 compared to WT. ## *p* < 0.01 compared to female. WT B: Wild type basal. WT PS: Wild type post-stress. TH-HZ B: TH-HZ basal. TH-HZ PS: TH-HZ post-stress.

**Figure 2:**
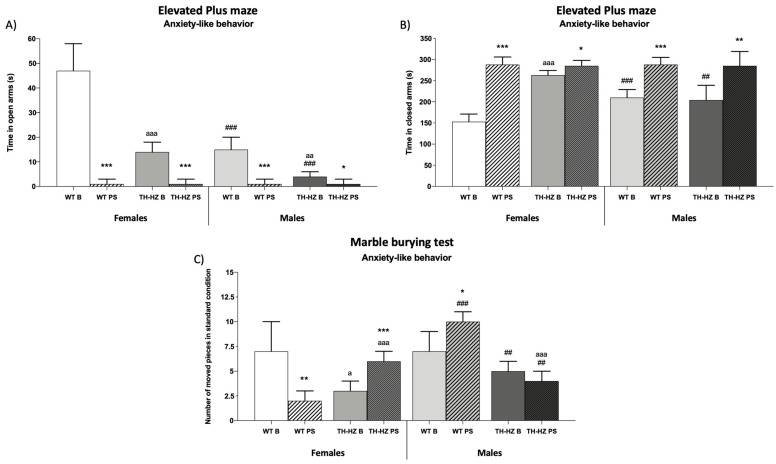
Anxiety-like behaviors. (**A**) Time (in seconds) in open arms of elevated plus maze (EPM). (**B**) Time (in seconds) in closed arms of EPM. (**C**) Number of pieces moved in the marble burying test. Each column represents the mean ± standard deviation of values corresponding to 6 animals. * *p* < 0.05, ** *p* < 0.01, *** *p* < 0.001 compared to basal condition, a *p* < 0.05, aa *p* < 0.01, aaa *p* < 0.001 compared to WT. ## *p* < 0.01, ### *p* < 0.001 compared to female. WT B: Wild type basal. WT PS: Wild type post-stress. TH-HZ B: TH-HZ basal. TH-HZ PS: TH-HZ post-stress.

**Figure 3:**
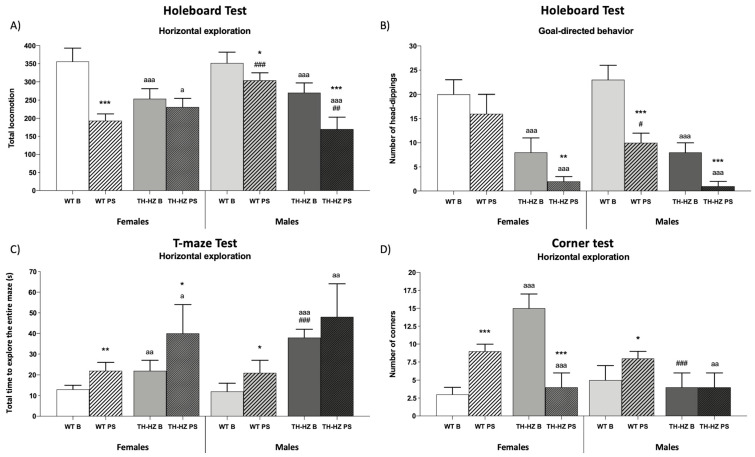
Exploratory behaviors. (**A**) Total locomotion in holeboard test (HBT). (**B**) Number of head-dippings performed in HBT. (**C**) Time (in seconds) to complete the entire T-maze. (**D**) Number of visited corners in the corner test. Each column represents the mean ± standard deviation of values corresponding to 6 animals. * *p* < 0.05, ** *p* < 0.01, *** *p* < 0.001 compared to basal condition, a *p* < 0.05, aa *p* < 0.01, aaa *p* < 0.001 compared to WT. # *p* < 0.05, ## *p* < 0.01, ### *p* < 0.001 compared to female. WT B: Wild type basal. WT PS: Wild type post-stress. TH-HZ B: TH-HZ basal. TH-HZ PS: TH-HZ post-stress.

To determine the differences between WT and TH-HZ females and males, and the effect of a punctual stress, we assayed in each experimental group (Female: WT Basal, WT Post-stress, TH-HZ Basal, and TH-HZ Post-stress; Male: WT Basal, WT Post-stress, TH-HZ Basal, and TH-HZ Post-stress) a battery of behavioral tests. This battery included: visual placing and hindlimb extensor reflex tests, wood rod test, a tightrope test, an elevated plus maze, a marble burying test, a holeboard test, a T-maze test, and a corner test. The results are summarized in Figure 1, Figure 2 and Figure 3 and Table 1 and Table 2.

#### 2.1.1. Sensorimotor Abilities

Related to the sensorimotor abilities, no differences were observed between TH-HZ and WT in basal condition (B) females except for in the tightrope test, where TH-HZ B females presented an elevated time of permanence (Table 1 *p* < 0.001), less latency to fall (Table 1 *p* < 0.001) and took more time to complete the test (Table 1 *p* < 0.01) than the WT B females group. In TH-HZ B males, more differences were observed compared to the WT B group. In the wood rod test, TH-HZ B males presented more latency to leave the first segment (Table 1 *p* < 0.01) and a greater time of permanence (Table 1 *p* < 0.001) than males of the WT B group, whereas in the tightrope test TH-HZ B males took more time of permanence (Table 1 *p* < 0.001), a higher percentage of individuals fell off (Table 1 *p* < 0.05), there was more latency to fall (Table 1 *p* < 0.001), and a lower percentage of individuals completed the test (Figure 1B *p* < 0.001) with the maximum traction (Figure 1C *p* < 0.01) compared with the male WT B group.

When the effects of a punctual stress were evaluated related to the sensorimotor abilities, it can be observed that WT females after stress (PS) performed the wood rod test worse than in those in the basal condition, who presented a higher time of permanence (Table 1 *p* < 0.05) and took more time for freezing (Table 1 *p* < 0.01). However, they performed the tightrope test better, as they spent less time to complete the test (Table 1 *p* < 0.05), showed a higher latency to fall (Table 1 *p* < 0.05), and had a better time of permanence (Table 1 *p* < 0.05) than females in the WT B group. Regarding TH-HZ PS females, they showed deteriorated sensorimotor abilities compared to the basal condition as they crossed fewer segments (Table 1 *p* < 0.05), took a greater time of permanence (Table 1 *p* < 0.05), less percentage of animals completed the test (Table 1 *p* < 0.01), and a greater percentage of animals performed freezing (Table 1 *p* < 0.01) during a greater time (Table 1 *p* < 0.01) in comparison to the female TH-HZ B group. Nevertheless, they took less time to complete the test (Figure 1A *p* < 0.05) than the TH-HZ B group. In the tightrope test, the TH-HZ PS group crossed fewer segments (Table 1 *p* < 0.01), a greater percentage of animals fell off (Table 1 *p* < 0.05), and any animal that completed the test (Figure 1B *p* < 0.001) as a lower percentage of animals performed the maximum traction (Figure 1C *p* < 0.05) compared with the TH-HZ B group. WT males after stress showed deteriorated sensorimotor abilities as they took a greater time of permanence (Table 1 *p* < 0.05) and more time to complete (Figure 1A *p* < 0.05) the wood rod test compared with males in the WT B group. TH-HZ PS males performed the wood rod test worse compared with the TH-HZ B group as they took a greater time of permanence (Table 1 *p* < 0.05), more time to complete the test (Figure 1A *p* < 0.05), and spent more time performing freezing (Table 1 *p* < 0.05), while they improved in the tightrope test as a higher percentage of animals completed the test (Figure 1B *p* < 0.001) with the maximum traction (Figure 1C *p* < 0.05) compared with TH-HZ B group.

Finally, when sex differences were considered, the male WT B group took less time to complete the wood rod test (Table 1 *p* < 0.01) and no one fell off the tightrope test (Table 1 *p* < 0.001) compared with the female WT B group. However, after punctual stress, WT males presented less latency to fall (Table 1 *p* < 0.01) and took more time to complete the tightrope test (Table 1 *p* < 0.01) than the female WT PS group. In the case of the TH-HZ group in basal conditions, males had a higher latency to fall (Table 1 *p* < 0.001), and no one completed the tightrope test (Table 1 *p* < 0.001) in comparison with the female TH-HZ B group. After stress, male TH-HZ mice took more time to complete the wood rod test (Figure 1A *p* < 0.01) and a higher percentage of animals completed the tightrope test (Figure 1B *p* < 0.01) compared with the female TH-HZ PS group.

#### 2.1.2. Anxiety-like Behaviors

When the anxiety-like behaviors were evaluated, in basal conditions the TH-HZ females presented higher anxiety levels compared with WT B female group. It was shown in the elevated plus maze, where TH-HZ B females spent less time in open arms (Figure 2A *p* < 0.001) and more time in closed arms (Figure 2B *p* < 0.001) compared with the WT B group. The male TH-HZ B group also showed higher anxiety levels compared with the WT B group, as they spent a smaller percentage of time in open arms (Table 1 *p* < 0.01). When the stress effects over anxiety-like behavior were evaluated, WT PS females presented higher anxiety levels, as they spent less time in open arms (Figure 2A *p* < 0.001) and more time in closed arms (Figure 2B *p* < 0.001) in the elevated plus maze compared to the female WT B group. In females, the TH-HZ PS group in the elevated plus maze revealed the same result as in the female WT group, thus, stress caused higher anxiety levels (Figure 2A *p* < 0.001; Figure 2B *p* < 0.05). However, in TH-HZ PS females, high anxiety levels were also reflected in the marble burying test, where they moved more pieces (Figure 2C *p* < 0.001) than the TH-HZ B group. On the other side, males after stress also presented higher anxiety levels. WT PS males spent less time in open arms (Figure 2A *p* < 0.001) and more time in closed arms (Figure 2B *p* < 0.001) in the elevated plus maze and moved more pieces in the marble burying test (Figure 2C *p* < 0.05) compared to the WT B group. Similarly, the male TH-HZ PS group showed the same results in the elevated plus maze (Figure 2A *p* < 0.05; Figure 2B *p* < 0.01) compared to the TH-HZ B group. When sex differences were analyzed, it could be observed that males showed higher anxiety levels than females. In the elevated plus maze, the male WT B and TH-HZ B groups spent less time in open arms (Figure 2A *p* < 0.001) and more time in closed arms (Figure 2B *p* < 0.001; *p* < 0.01) compared with the female WT B and TH-HZ B groups, respectively. After the stress condition, in the elevated plus maze, either the WT PS or TH-HZ PS males presented higher anxiety levels compared with their corresponding female groups. In contrast, in the marble burying test WT PS males moved more pieces in standard condition (Figure 2C *p* < 0.001), while TH-HZ PS males moved fewer pieces in standard condition (Figure 2C *p* < 0.01) in comparison to the females of each group.

#### 2.1.3. Exploratory Behaviors

Regarding exploratory behaviors, TH-HZ B females exhibited an impaired exploration compared with the female WT B group. It was shown in the holeboard test (HBT), where TH-HZ B mice presented a lower inner, middle, and total locomotion (Table 2 and Figure 3A *p* < 0.001), and a lower number of head-dippings (Figure 3B *p* < 0.001). In the T-maze test, they took more time to complete the test (Figure 3C *p* < 0.01) compared to the female WT B group. TH-HZ B males showed similar results as TH-HZ B females. They presented a lower inner, middle, and total locomotion (Table 2 *p* < 0.01; *p* < 0.001; Figure 3A *p* < 0.001), and a lower number of head-dippings (Figure 3B *p* < 0.001) in HBT, and they spent more time completing the T-maze test (Figure 3C *p* < 0.001) compared to the male WT B group.

In WT females, after stress lower locomotion was observed in HBT. In fact, this was observed in the total (Figure 3A *p* < 0.001) and in the external, middle, and inner areas (Table 2 *p* < 0.05; *p* < 0.01; *p* < 0.01) as well as in the T-maze, where they took more time to complete the test (Figure 3C *p* < 0.01), whereas in the corner test it was observed that they visited more corners (Figure 3D *p* < 0.001) in comparison to the female WT B group. After stress, TH-HZ females showed a lower inner and external locomotion (Table 2 *p* < 0.01), as well as lower goal-directed exploration as they performed fewer head-dippings (Figure 3B *p* < 0.01) in HBT compared with the TH-HZ B group. They also took more time to complete the T-maze test (Figure 3C *p* < 0.05) and visited a smaller number of corners (Figure 3D *p* < 0.001) in the corner test with respect to the TH-HZ B group. Similarly, males after stress showed deteriorated exploratory behavior. WT PS males performed lower inner, middle, and total locomotion (Table 2 *p* < 0.001; Figure 3A *p* < 0.05) as well as a smaller number of head-dipping (Figure 3B *p* < 0.001) in HBT and spent more time to complete the T-maze test (Figure 3C *p* < 0.05), whereas in the corner test, they visited more corners (Figure 3D *p* < 0.05) in comparison to the WT B group. TH-HZ PS males also showed lower locomotion (Table 2 *p* < 0.001; Figure 3A *p* < 0.001) and a deteriorated goal-directed exploration (Figure 3B *p* < 0.001) in HBT compared to the TH-HZ B group.

Sex differences in exploratory behaviors were also evident. It was observed that WT B males presented lower locomotion (Table 2 *p* < 0.05) in HBT compared with WT B females, and this trend was also shown by WT PS males (Table 2 *p* < 0.001) compared to WT PS females. TH-HZ B males took more time to complete the T-maze (Figure 3C *p* < 0.001) and visited a fewer number of corners (Figure 3D *p* < 0.001) in the corner test in comparison to the TH-HZ B females. After stress conditions, TH-HZ males showed lower total locomotion (Figure 3A *p* < 0.01) in HBT compared with TH-HZ PS female group.

### 2.2. Immune Function

**Figure 4:**
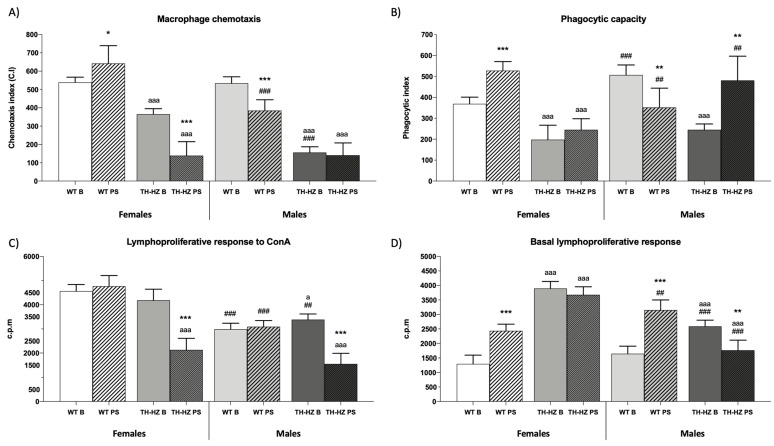
Immune function parameters. (**A**) Macrophage chemotaxis index. (**B**) Phagocytic index. (**C**) Lymphoproliferative response to ConA (c.p.m). (**D**) Basal lymphoproliferative response (c.p.m). Each column represents the mean ± standard deviation of values corresponding to 6 animals. * *p* < 0.05, ** *p* < 0.01, *** *p* < 0.001 compared to basal condition, a *p* < 0.05, aaa *p* < 0.001 compared to WT. ## *p* < 0.01, ### *p* < 0.001 compared to female. WT B: Wild type basal. WT PS: Wild type post-stress. TH-HZ B: TH-HZ basal. TH-HZ PS: TH-HZ post-stress.

When the immune function parameters were evaluated, several differences were observed. TH-HZ B females and males presented impaired immunity in comparison with both the WT B groups. These impairments are reflected in macrophage functions as the chemotaxis (Figure 4A *p* < 0.001), phagocytic efficacy (Table 3 *p* < 0.001), and phagocytic index (Figure 4B *p* < 0.001), as well as in lymphocyte functions as chemotaxis (Table 3 *p* < 0.001), basal and LPS proliferative responses (Figure 4D *p* < 0.001; Table 3 *p* < 0.001), and in the antitumoral natural killer activity (Table 3 *p* < 0.001). After stress, WT females improved their macrophage chemotaxis (Figure 4A *p* < 0.05), phagocytic efficacy (Table 3 *p* < 0.01), phagocytic index (Figure 4B *p* < 0.001), and their natural killer activity (Table 3 *p* < 0.001), whereas lymphocyte chemotaxis (Table 3 *p* < 0.001) and basal lymphoproliferation (Figure 4D *p* < 0.001) were impaired compared to the WT B group. TH-HZ females did not show any improvement after stress; thus they presented an impaired macrophage and lymphocyte chemotaxis (Figure 4A *p* < 0.001; Table 3 *p* < 0.001), and lymphoproliferation in response to ConA and LPS (Figure 4C *p* < 0.001; Table 3 *p* < 0.001) in comparison to the TH-HZ B group. Similarly, males after stress showed a general impairment of their immune function. WT PS males showed an altered macrophage function in their chemotaxis (Figure 4A *p* < 0.001), phagocytic efficacy (Table 3 *p* < 0.05), and phagocytic index (Figure 4B *p* < 0.01), and a higher basal lymphoproliferation (Figure 4D *p* < 0.001) compared to the WT B group. On their side, TH-HZ PS males showed greater impairment in their phagocytic efficacy (Table 3 *p* < 0.01), phagocytic index (Figure 4B *p* < 0.01), lymphocyte chemotaxis (Table 3 *p* < 0.05), a higher basal lymphoproliferation (Figure 4D *p* < 0.01), a lower lymphoproliferation in response to ConA and LPS (Figure 4C *p* < 0.001; Table 3 *p* < 0.001), and a deteriorated natural killer activity (Table 3 *p* < 0.01) with respect to the TH-HZ B group. In general, males of all groups presented an impaired immune function compared to females of the same group (Table 3 and Figure 4).

### 2.3. Oxidative Stress

**Figure 5:**
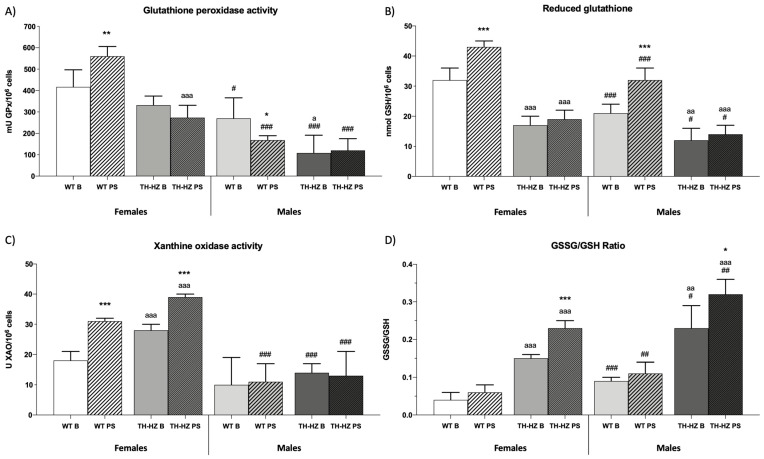
Oxidative stress parameters. (**A**) Glutathione peroxidase activity (mU GPx/10^6^ cells). (**B**) Reduced glutathione levels (nmol GSH/10^6^ cells). (**C**) Xantine oxidase activity (U XO/10^6^ cells) (**D**) GSSG/GSH ratio. Each column represents the mean ± standard deviation of values corresponding to 6 animals. * *p* < 0.05, ** *p* < 0.01, *** *p* < 0.001 compared to basal condition, a *p* < 0.05, aa *p* < 0.01, aaa *p* < 0.001 compared to WT. # *p* < 0.05, ## *p* < 0.01, ### *p* < 0.001 compared to female. WT B: Wild type basal. WT PS: Wild type post-stress. TH-HZ B: TH-HZ basal. TH-HZ PS: TH-HZ post-stress.

When the oxidative stress parameters were evaluated, TH-HZ B females exhibited a greater rate of oxidative stress in comparison to the WT B group. They showed lower catalase (CAT) and glutathione reductase (GR) activities (Table 3 *p* < 0.001; *p* < 0.05), lower levels of reduced glutathione (GSH) (Figure 5B *p* < 0.001), higher xanthine oxidase (XO) activity (Figure 5C *p* < 0.001), higher levels of oxidized glutathione (GSSG) (Table 3 *p* < 0.05), and a higher GSSG/GSH ratio (Figure 5D *p* < 0.001) with respect to the female WT B group. TH-HZ B males presented lower amounts of antioxidant defenses, such as GR (Table 3 *p* < 0.01) and glutathione peroxidase (GPx) activities (Figure 5A *p* < 0.05), as well as lower amounts of GSH (Figure 5B *p* < 0.01) and higher levels of oxidant compounds such as GSSG (Table 3 *p* < 0.001), which were translated in a higher GSSG/GSH ratio (Figure 5D *p* < 0.01) in comparison to the WT B group. After stress, WT females showed greater antioxidant defenses, GR, and GPx activities (Table 3 *p* < 0.001; Figure 5A *p* < 0.01), and higher levels of GSH (Figure 5B *p* < 0.001), but they also exhibited greater amounts of oxidant compounds as evidenced by the XO activity and GSSG (Figure 5C *p* < 0.001; Table 3 *p* < 0.001) compared to the WT B group. Female TH-HZ PS mice presented greater amounts of GR and XO activity, and GSSG (Table 3 *p* < 0.05; Figure 5C *p* < 0.001; Table 3 *p* < 0.001;), as well as a higher GSSG/GSH ratio (Figure 5D *p* < 0.001) with respect to the TH-HZ B group. Males after stress showed a similar response to females. WT PS males exhibited higher levels of CAT, GR activities, and GSH (Table 3 *p* < 0.01; *p* < 0.05; Figure 5B *p* < 0.001), lower GPx activity (Figure 5A *p* < 0.05), and higher amounts of GSSG (Table 3 *p* < 0.001) compared to the WT B group. TH-HZ PS males showed a greater GR activity (Table 3 *p* < 0.001), as well as greater levels of GSSG (Table 3 *p* < 0.001) and a higher GSSG/GSH ratio (Figure 5D *p* < 0.05) with respect to the TH-HZ B group. Finally, when females and males were compared, it was observed that males presented a higher oxidative profile than females, having lower levels of antioxidant compounds and greater amounts of oxidant compounds in comparison to females, and this was reproduced in all experimental groups (Table 3 and Figure 5).

### 2.4. Catecholamine Concentrations

**Figure 6:**
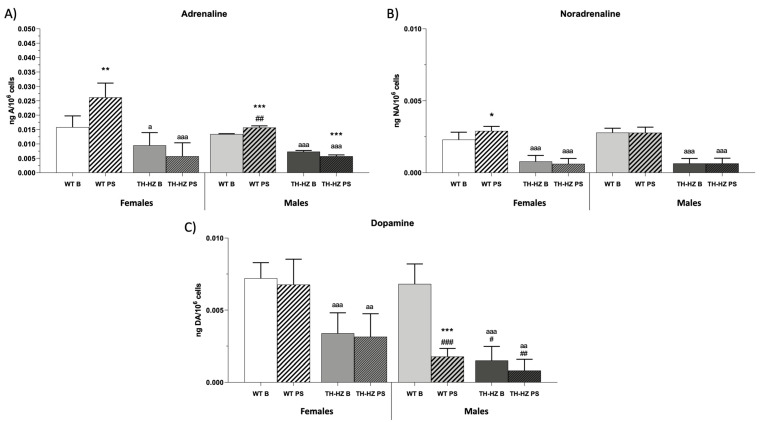
Catecholamine concentrations. (**A**) Adrenaline (ng A/10^6^ cells). (**B**) Noradrenaline (ng NA/10^6^ cells). (**C**) Dopamine (ng DA/10^6^ cells). Each column represents the mean ± standard deviation of values corresponding to 6 animals. * *p* < 0.05, ** *p* < 0.01, *** *p* < 0.001 compared to basal condition, a *p* < 0.05, aa *p* < 0.01, aaa *p* < 0.001 compared to WT. # *p* < 0.05, ## *p* < 0.01, ### *p* < 0.001 compared to female. WT B: Wild type basal. WT PS: Wild type post-stress. TH-HZ B: TH-HZ basal. TH-HZ PS: TH-HZ post-stress.

Finally, the catecholamine concentrations were evaluated. TH-HZ B females and males showed lower concentrations of adrenaline (A) (Figure 6A *p* < 0.05; *p* < 0.001), noradrenaline (NA) (Figure 6B *p* < 0.001; *p* < 0.001), and dopamine (DA) (Figure 6C *p* < 0.001; *p* < 0.001) in their peritoneal leukocytes compared to the WT B group. After stress, female WT PS mice increased their A and NA concentrations (Figure 6A *p* < 0.01; Figure 6B *p* < 0.05) with respect to the female WT B group, whereas the male WT PS group increased their A amounts (Figure 6A *p* < 0.001) and decreased their DA concentrations (Figure 6C *p* < 0.001) in comparison to the male WT B group. Regarding the TH-HZ groups, no differences were observed after stress, except for a decrease in the A concentrations in the male TH-HZ PS group (Figure 6A *p* < 0.001). Finally, in basal conditions, there was not any difference due to sex in the WT group, but after stress WT PS males showed a lower concentration of A and DA (Figure 6A *p* < 0.01; Figure 6C *p* < 0.001) with respect to the female WT PS group. In the TH-HZ group, the catecholamine concentrations were similar in females and males. Only TH-HZ males showed a lower concentration of DA in the basal and post-stress conditions (Figure 6A *p* < 0.05; Figure 6A *p* < 0.01) with respect to the female TH-HZ groups.

## 3. Discussion

This study is the first to analyze the effects that a punctual stress can have on the organism both under normal conditions and when catecholamine synthesis is affected by a genetic haploinsufficiency, clarifying the importance of catecholamine synthesis in the acute stress response in vivo. In addition, it has also allowed us to know the different responses to a punctual stress due to sex.

Firstly, the differences between wild type (WT) and hemizygotes for the tyrosine hydroxylase (TH-HZ) genotype were confirmed by observing the results obtained in basal conditions and comparing these ones with the results previously published using these same animals. In fact, both female and male TH-HZ mice presented impaired sensorimotor skills and exploratory behaviors, as well as higher levels of anxiety compared to the WT ones. In addition, both innate and adaptive immunity as well as the redox state of TH-HZ mice were altered, showing a pro-inflammatory and pro-oxidant profile. These results confirm the previous ones shown by Garrido and colleagues [4,15,17,25,26], where the authors described that female mice at adult age with this genetic haploinsufficiency constitute a model of premature aging in mice, since all their homeostatic systems (nervous, immune, and endocrine) are impaired, together with a shorter lifespan. 

It is known that catecholamine synthesis is essential for providing an adequate response to any stressful situation, and there are many studies that investigate how they act in the face of different stressors, their route of action, and the different responses of the organism in the face of acute or chronic stress [18,19,20,21,22,23]. However, few of them focus on studying how the response to a stressor would be when the catecholamine synthesis is reduced. In our case, we wanted to observe how a punctual stress by a 10 min immobilization affected both WT and TH-HZ mice.

In the case of WT mice, we observed that after this punctual stress, the sensorimotor abilities of the animals did not suffer any change, while the anxiety levels were increased and exploratory abilities were reduced, compared to their basal condition. This could be due to the effect of immobilization with the restraint, as it has been observed how impeding the movement of the animals produces elevated anxiety levels [27]. 

Regarding the immune function, it was observed how WT PS females improved their innate response by presenting higher macrophage chemotaxis and higher phagocytic efficiency, as well as better Natural Killer activity. On the contrary, WT PS males showed an impairment of their innate immune function. This sex difference would be due to the fact that the final release of glucocorticoids and corticosteroids is highly influenced by gonadal hormones and genes on the sex chromosome [24]. With regard to adaptive functions, there are no differences in any WT group that were obtained, which could be due to the type of stress, since, being a punctual stress, it would not be so prolonged as to intervene with the adaptive immunity [28,29]. What could be observed in WT PS mice was an increased sterile inflammation after stress. Something similar to this can be observed in the oxidative stress parameters, where it can be observed how WT PS females, despite an increase in their oxidative compounds (oxidized glutathione and xanthine oxidase), that the punctual stress also produced a favorable response in their antioxidant defenses (reduced glutathione, glutathione reductase, and peroxidase) that allowed the organism to maintain a balance in its oxidative state. Similarly, WT PS males also managed to maintain oxidative balance through an increased reduced glutathione and glutathione reductase activity, despite increased oxidized glutathione. Maintaining oxidative balance is essential for the proper functioning of homeostatic systems and thus for the maintenance of health [4].

In the case of TH-HZ mice, the response to the punctual stress was worse than in WT. This is to be expected since, presenting a reduced synthesis of catecholamines, they cannot perform an adequate response to this stressful situation [15,25]. Firstly, it can be observed how the nervous function was impaired after stress, resulting in an impaired equilibrium and neuromuscular vigor, as well as in elevated levels of anxiety and decreased directed and undirected exploratory abilities compared to TH-HZ B group. Similarly, both the innate and adaptive immune function were either impaired or remained the same as in the basal condition. This is logical considering that already in the basal condition these functions were quite reduced compared to their WT B controls. However, it is probable that if the stressor had been prolonged over time, the immune function in the TH-HZ group would have deteriorated, compromising the health of these individuals [28,29]. Regarding the oxidative state, it is worth mentioning that the TH-HZ PS group failed to regulate the increase in oxidants that they underwent with stress (oxidized glutathione and xanthine oxidase activity), establishing a clear oxidative stress as indicated by the GSSG/GSH ratio [30].

This study, beyond the differences observed between the different genotypes and the response to stress, has also revealed the differences that exist at the nervous, immune, and oxidative state levels simply because they belong to one sex. In WT, it can be observed how females presented generally better behavioral abilities than males by having better equilibriums and lower anxiety, while in exploration they behaved the same, as already observed in other studies [31,32,33]. Regarding the innate immunity, both sexes presented similar functionality in the basal condition, while in adaptive immunity it can be observed how the males presented a worse proliferative response to mitogens, showing a worse inflammatory response than females [34]. It is worth noting that the males in basal conditions were much more oxidized than females as the GSSG/GSH ratio shows us, mainly due to a lower antioxidant response [35], and to the fact that their mitochondria produce twice as many ROSs as those of females [36]. Finally, it should be noted that WT males had a worse response to stress than WT females, as they exhibited greater nervousness in their behavior that could sometimes lead to the misinterpretation of results, as well as impaired innate and adaptive immune functionality, a greater sterile inflammation and, of course, a situation of oxidative stress higher to that of females. This fact may be mainly due to a condition associated with gonadal hormones and sex chromosomes, which act at different levels of the HPA axis [37]. This poorer adaptive capacity of males would be one of the reasons why they have a lower longevity [38]. The same is shown in the case of the TH-HZ mice, however, in this genotype, the differences due to sex are not as notable as in the WT. This may be due to the fact that, regardless of sex, the TH-HZ mice start with having impaired nervous, immune, and oxidative functions compared to WT mice, so that, as mentioned above, there is a limit below which a functional response would not occur, so that the difference between this limit and the impaired functionality presented by TH-HZ mice cannot be as wide as that observed in WT mice.

Finally, the catecholamine deficit in TH-HZ mice has been constantly discussed, and this study points to how the different catecholamine synthesis affects the stress response in the different experimental groups. After stress, WT females increased their adrenaline and noradrenaline concentration in peritoneal leukocytes, while the males showed an increase in their adrenaline concentration and a decrease in dopamine concentration. In TH-HZ mice, it can be observed that after stress there was no increase in the concentration of any catecholamine; moreover, adrenaline decreased in males. Finally, with respect to sex, WT males in basal conditions did not show any difference in their catecholamine concentration compared to females, while after stress males showed a lower concentration of adrenaline and dopamine than females. In addition, TH-HZ males, independently of the punctual stress showed no difference in adrenaline and noradrenaline concentration with respect to TH-HZ females, while, both in the basal and post-stress condition, they presented a lower concentration of dopamine. 

These differences between the experimental groups in their catecholamine concentrations explain the results obtained when the different functions are evaluated given the close relationship between catecholamines and the homeostatic systems [7,39]. Regarding immunity, stress and catecholamine release regulate many functions of the immune system, such as cytokine production [40,41], proliferation [42], apoptosis of splenic cells [43], changes in leukocyte subsets [44,45], splenic macrophage phagocytosis [46], and NK cell cytotoxicity [12], thus catecholamines can be said to play a key role in the regulation of innate and adaptive immunity [13]. In this sense, the regulation of the immune system is mainly mediated by noradrenaline and dopamine, moreover, the immune cells themselves are able to synthesize noradrenaline through dopamine beta-hydroxylase [13], which could justify the decrease in this in WT males after stress, since they would be dealing dopamine to the synthesis of noradrenaline, trying to maintain their immune function. In addition, the increase in noradrenaline that occurs in WT females after stress would be favoring the improvement of the immune system functionality. In addition, an increase in adrenaline has been related to an increased production of proinflammatory cytokines [47], which would explain the increase in sterile inflammation observed in our study. Furthermore, as the immune system is closely related to the oxidative state of individuals [4,5], this leads to noradrenaline and dopamine also playing a role in the maintenance of the redox state. Based on this, it is known that these two hormones have antioxidant properties [48], which agrees with the results obtained, since we observed that WT PS females increased the concentration of noradrenaline that favors the action of antioxidant defenses, such as glutathione peroxidase and glutathione reductase activity that aim to produce reduced glutathione to balance the oxidative compounds produced, among others, by xanthine oxidase. In the case of WT males, we observed that after stress, there was no increase in noradrenaline, but they were able to maintain redox balance compared to the basal condition. Finally, when we look at TH-HZ mice, it can be observed how a clear oxidative state was established after stress in both males and females. This may be because the stress is indeed inducing an oxidative response, however, the antioxidant response is not occurring, which may be due in part to the non-synthesis of noradrenaline and dopamine. Furthermore, the establishment of this oxidative state in leukocytes due to stress agrees with the results observed by other authors, where stress was related to oxidative damage to leukocyte DNA [49]. These results highlight the regulatory role of catecholamines in the stress response. However, there may be other mediators acting in the stress response, such as the 5-hydroxytryptamine (5-HT), and different neuropeptides including endogenous opioids or cholecystokinin (CCK), for which there is evidence of the regulation of stress [50,51], and which would be interesting to evaluate in future studies.

It is worth mentioning that stress and how it is managed is somewhat complex since each individual perceives the same stressful stimulus in a different way [52]. Therefore, the terms eustress and distress are increasingly coined, the first providing a beneficial response for the organism and the second one a response that compromises the health of the organism [2]. Thus, several studies report different responses to the same stressor agent that agree that the greater the perceived stress is, for instance, whether it involves distress, the immune function will be impaired, and a redox state will be established, leading to a deterioration of the homeostatic systems and, therefore, to a loss of health and shorter longevity [52,53].

In our case, we can conclude that after a punctual stress, WT mice are able to provide an adequate response to the punctual stress induced by restraint, this response being different depending on the sex. However, TH-HZ mice, given their limited catecholamine synthesis, are unable to provide an adequate response to stress, regardless of sex.

## 4. Materials and Methods

### 4.1. Animals

For this study, we used adult (9 ± 1 month) virgin female and male TH-HZ and wild type (WT) ICR-CD1 mice. These animals were derived from a colony from the laboratory of Dr. Flora de Pablo as previously described [54]. Although these TH-HZ mice contain only one tyrosine hydroxylase allele, they stay healthy and normal with no signs of any associated lesions. In addition, growth rates were indistinguishable from those of WT animals. They were housed 6 per cage, separated by sex and genotype, and maintained with ad libitum access to food and tap water under light (12/12 h reversed light/dark cycle; lights off at 8:00 A.M.) to avoid circadian interferences. The temperature (22 ± 2 °C) and humidity (50–60%) were also controlled. The diet was in accordance with the recommendations of the American Institute of Nutrition for laboratory animals (A04 diet from Panlab S.L., Barcelona, Spain). Experiments were performed during the dark phase of the cycle (8:00–12:00 h). The protocol was approved by the Experimental Animal Committee of Complutense University of Madrid (Spain) (PROEX 224.0/21). The animals were treated according to the guidelines of the European Community Council Directives ECC/566/2015.

### 4.2. Experimental Design

The animals were divided into the following groups: female TH-HZ (n = 6), WT (n = 6), and male TH-HZ (n = 6), WT (n = 6). All experimental groups were submitted to a battery of behavioral tests, evaluating their sensorimotor abilities, anxiety-like behaviors, and exploratory capacities. After that, their peritoneal leukocytes were extracted and several immune functions, as well as the oxidative stress parameters and the catecholamines concentration, were assessed. These analyses were considered the basal condition. Then, the mice were subjected to punctual restriction stress (10 min), and they were exposed to the same behavioral tests, and then the same immune and oxidative stress parameters were assessed after restriction (post-stress condition).

### 4.3. Restriction Stress

Mice were subjected to punctual restriction stress for 10 min in a cylindrical, transparent, acrylic tank (height = 8.5 cm, diameter = 2.5 cm) fixed on a square pedestal. The diameter of the cylinder was made to fit the body, avoiding the movement of the mouse. Adequate ventilation was provided using holes at the sides of the tube.

### 4.4. Behavioral Tests

Behavioral testing took place for five consecutive days. The tests and sequence were chosen based on previous reports [15,55]. On the first day, the animals were subjected to the whole battery of sensorimotor abilities, T-maze, and corner tests. On the second and third days, they were exposed to the holeboard test and the elevated plus maze, respectively. After that, the animals were isolated for 24 h and on the fourth and fifth days they performed the marble burying test. The tests were carried out under red light with a white light lamp (20 W) and were started by placing the animals in the area of the apparatus considered most behaviorally neutral so that the mouse was not artificially induced to perform a significant pattern [56]. The apparatuses were cleaned with 70% ethanol between animals to avoid possible olfactory interferences.

#### 4.4.1. Sensorimotor Abilities

- Visual Placing and Hindlimb extensor reflex

This test was performed as previously described [57]. For this, mice were suspended by the tail and lowered toward a black surface. Complete extension of their forelimbs and hindlimbs was considered a positive response. The response was measured in three trials and the mean value was calculated.

- Wood rod test

To assess their motor coordination and equilibrium, the animals were placed in the center of a 2.9-cm wide and 80-cm long wooden rod, being suspended in the air 22 cm with the help of two bases and divided into 10 cm segments. Motor coordination was measured by the latency to leave the starting segment, the total number of crossed segments, and time of permanence, while the equilibrium was measured by the time taken to finish the trial, the percentage of animals falling off the rod, as well as the time taken to fall [57]. Other behaviors such as freezings (number, time in seconds, and percentage of animals performing freezings) were also recorded.

- Tightrope test

The tightrope test was developed to evaluate motor coordination, muscular vigor, and traction [58]. The device consists of a 60-cm long tightrope divided into 10-cm segments and elevated 40 cm high, which is held by two metallic rods. For this, the mice were hung by their forelimbs in the middle of the tightrope. Motor coordination was evaluated by the total number of segments crossed and by the time of permanence (in seconds). Muscular vigor was evaluated by the percentage of mice that fell off, the latency (seconds) to fall, the percentage of mice that complete the test, as well as the time (seconds) to complete the test. Finally, traction was evaluated by analyzing the different parts of the body that the mice used to keep hanging (forelimbs, hindlimbs, and tail). The percentage of mice displaying low (forelimbs), medium (forelimbs and hindlimbs), and maximum traction (forelimbs, hindlimbs, and tail) were also analyzed.

#### 4.4.2. Exploratory Behavioral Tests

- T-Maze test

This test is used to evaluate the spontaneous horizontal exploratory behavior [57,59]. The device consists of a T-shaped maze (short arms: 25 × 10 cm, long arm: 65 × 10 cm, walls: 20 cm high). Mice were placed in the short arm facing the wall. The time (seconds) spent crossing the intersection and the time (seconds) spent exploring the entire maze as parameters of the horizontal exploration, and the number of rearings, and the time (seconds) of each rearing as parameters of the vertical exploration were recorded. Other behaviors such as groomings and freezings (number and time in seconds) were also considered.

- Corner test

This test was used to evaluate the spontaneous horizontal exploratory behavior [15]. For this, a square cage (22 cm) was used. The duration of the test was 30 s, in which the number of visited corners, wall rearings, groomings, and scratches were analyzed.

- Holeboard test

To analyze the non-goal-directed behavior (evaluated by horizontal and vertical activity), as well as the goal-directed behavior (evaluated by the number and time of head-dipping), mice performed the holeboard test. The device consists of a box (60 × 60 × 45) divided into 36 squares (10 × 10 cm), with four equidistant holes (3.8 cm diameter) in the inner zone. We considered the inner zone as the four central squares, the external zone as the 20 squares nearest the walls, and the 12 remaining squares as the middle zone. In each hole, plastic objects were placed to attract the animal’s attention and drive their goal-directed behavior. The duration of the test was 5 min [15,57], and during this time the parameters for non-goal-directed and goal-directed behavior were recorded. For non-goal-directed behavior, we evaluated total, external, middle, and inner locomotion, the average of all of them (the number of crossed squares in each area divided by the number of squares that conform that area), and the percentage of all of them (the number of crossed squares in each area divided by the total locomotion). All these parameters were considered horizontal activity. Regarding the vertical activity, the number of the wall- and central rearing, and the time (seconds) of each rearing were recorded. Finally, for goal-directed behavior, the total number of head-dipping and the time (seconds) of each head-dipping were evaluated. Other behaviors such as grooming and freezing (number and time in seconds) were also considered.

#### 4.4.3. Anxiety Behavioral Tests

- Elevated plus maze

Anxiety levels were evaluated by the elevated plus maze test [60]. The device consists of two open arms (45 × 10 cm) and two closed arms (5 × 10 × 50 cm) that extend from a central platform (10 × 10 cm), elevated 65 cm above the floor. The duration of the test was 5 min. Mice were placed on the central platform facing a closed arm, and the total number of entries in the open and closed arms, the time spent (seconds) in the central platform, the time spent in the open and closed arms, and the percentage of time in the central platform and open and closed arms (time spent in each area divided by the total time) were evaluated. Other behaviors such as grooming, and rearing (number and time in seconds) were also recorded.

- Marble Burying test

The burying behavior in rodents reflects their ability to interact with the environment [61]. For this analysis, the animals were habituated to isolation 24 h before performing the test. The test was divided into two conditions: standard and bizonal condition. For the standard condition, 12 marbles were placed along the cage and, after 15 min, the number of moved, intact, and buried marbles were counted. A total of 24 h after performing in the standard condition, the mice were submitted to the bizonal condition. In this case, 8 marbles were placed in one half of the cage, with the remaining other half free and, after 20 min, the number of moved, intact, and buried marbles were counted.

### 4.5. Collection of Peritoneal Leukocytes

Murine peritoneal leukocytes were collected between 9:00 and 12:00 h a.m. to avoid differences due to circadian variations. The mice were immobilized by cervical skin, and 3 mL of Hank’s solution at 37 °C was injected into their peritoneal cavity [62]. After the abdomen massage, approximately 80% of Hank’s solution enriched with peritoneal leukocytes was recovered. Then, macrophages and lymphocytes identified by their morphology, were quantified in a Neubauer chamber. The cellular viability was measured with the Trypan-blue (Sigma-Aldrich, St. Louis, MO, USA) exclusion test, and it was higher than 98% in all cases. Peritoneal suspensions were adjusted to a specific number of macrophages, lymphocytes, or total leukocytes, depending on the analyzed parameter, as described in the corresponding section.

### 4.6. Immune Function Parameters

- Chemotaxis

The chemotaxis capacity of peritoneal leukocytes was determined according to Boyden’s method with modifications introduced by our group [63]. It is based on the immune cell capacity to migrate to an infectious focus. Cell suspensions were adjusted to 0.5 × 10^6^ macrophages or lymphocytes/mL in Hank’s solution and placed into the upper compartment of the Boyden’s chamber, and f-met-leu-phe (Sigma, St. Louis, MO, USA) (a positive chemotactic peptide in vitro) was placed in the lower compartment. After a 3 h incubation, the filters were fixed and stained with Giemsa (Sigma-Aldrich). Finally, the chemotaxis index (C.I.) was determined by counting the total number of macrophages or lymphocytes on one-third of the lower face of the filters.

- Macrophage phagocytosis

The phagocytic capacity of peritoneal cells was evaluated as previously described [63,64]. This protocol is based on the macrophage’s capacity to ingest inert particles (latex beads). Cell suspensions were adjusted to 0.5 × 10^6^ macrophages/mL in Hank’s solution and placed into migration inhibitor factor (MIF)-coated plates for 30 min. Then, latex beads were added to the adherent cell monolayer. After 30 min of incubation, the plates were washed, fixed, and stained with Giemsa (Sigma-Aldrich). Finally, the number of latex beads ingested by 100 macrophages (phagocytic index) and the number of macrophages that ingest at least one latex bead (phagocytic efficacy) were determined.

- Natural Killer Activity

This was evaluated as previously described [65]. Cell suspensions were adjusted to 10^6^ peritoneal leukocytes/mL in RPMI 1640 medium and placed into 96-well U-bottom plates. Murine YAC-1 lymphoma cells were added into wells, and the NK activity was assessed by quantifying the released lactate dehydrogenase into the medium (Cytotox 96 TM, Promega, Stuttgart, Germany). The results were expressed as the percentage of tumor cells killed (% lysis), as previously described [63].

- Lymphoproliferative capacity

This was evaluated as previously described [62,63]. Spontaneous lymphoproliferation, as well as in response to concanavalin A (1 μg/mL ConA; Sigma-Aldrich) and lipopolysaccharide (1 μg/mL LPS, *Escherichia coli*, 055: B5; Sigma-Aldrich), were evaluated. For this, cell suspensions were adjusted to 0.5 × 10^6^ lymphocytes/mL in RPMI 1640 medium supplemented with gentamicin and fetal bovine serum (FBS) and placed into 96-well plates. After 48 h of incubation at 37 °C in a sterile and humidified atmosphere of 5% CO_2_, ^3^H-thymidine was added and incubated for 24 h. Finally, the cells were collected in a semi-automatic harvester, and ^3^H-thymidine uptake was quantified in a beta counter. The results were expressed in counts per minute (c.p.m).

### 4.7. Oxidative Stress Parameters

- Catalase activity

Cellular suspensions were adjusted to 10^6^ leukocytes/mL in Hank’s solution, centrifugated, and the cell pellets were resuspended in a 50 mM oxygen-free phosphate buffer. Then, they were sonicated, and supernatants were used for the enzymatic reaction with 14 mM H_2_O_2_ as substrate. The enzymatic assay was followed spectrophotometrically for 80 s at 240 nm by the decomposition of H_2_O_2_ into H_2_O + O_2_ as previously described [66]. The results were expressed in International Units (IU) of enzymatic activity per 10^6^ peritoneal leukocytes.

- Glutathione reductase activity

Cellular suspensions were adjusted to 10^6^ leukocytes/mL in Hank’s solution, centrifugated, and the cell pellets were resuspended in 50 mM oxygen-free phosphate buffer with 6.3 mM EDTA. Then, they were sonicated, and supernatants were used for the enzymatic reaction with 80 mM GSSG 80 as a substrate. The oxidation of NADPH was followed spectrophotometrically by the decrease in the absorbance at 340 nm for 240 s as previously described [66]. The results were expressed in mU of enzymatic activity per 10^6^ peritoneal leukocytes.

- Glutathione peroxidase activity

Cellular suspensions were adjusted to 10^6^ leukocytes/mL in Hank’s solution, centrifugated, and the cell pellets were resuspended in 50 mM oxygen-free phosphate buffer. Then, they were sonicated, and supernatants were used for the enzymatic reaction with cumene hydroperoxide as a substrate (cumene-OOH). The oxidation of NADPH was followed spectrophotometrically by the decrease in the absorbance at 340 nm for 300 s as previously described [66]. The results were expressed in mU of enzymatic activity per 10^6^ peritoneal leukocytes.

- Glutathione concentration

Cellular suspensions were adjusted to 10^6^ leukocytes/mL in Hank’s solution, centrifugated, and the cell pellets were resuspended in 50 mM phosphate buffer with 0.1 EDTA, pH 8. Then, they were sonicated, and supernatants were used for the quantification of both reduced (GSH) and oxidized (GSSG) glutathione by the reaction capacity that GSSG and GSH have with o-phthalaldehyde (OPT) at pH 12 and pH 8, respectively, resulting in the formation of a fluorescent compound. The fluorescence was measured at 350 nm excitation and 420 nm emission, as previously described [15]. The results were expressed in nmol of GSSG and GSH per 10^6^ peritoneal leukocytes. Moreover, the GSSG/GSH ratio was calculated for each sample.

- Xanthine oxidase activity

Xanthine oxidase (XO) activity was assayed using a commercial kit (A-22182 Amplex Red Xanthine/Xanthine Oxidase Assay Kit, Molecular Probes, Paisley, UK). Cellular suspensions were adjusted to 10^6^ leukocytes/mL in Hank’s solution, centrifugated, and the cell pellets were resuspended in 50 mM potassium phosphate buffer with 0.1 M EDTA and 0.5 mM DTT. Supernatants were used for the enzymatic reaction with the working solution of the Amplex Red reagent. The fluorescence was measured at 530 nm excitation and 595 nm emission. The results were expressed in units (U) of enzymatic activity per 10^6^ peritoneal leukocytes.

### 4.8. Concentration of Catecholamines

The concentrations of catecholamines were assessed in peritoneal leukocytes using the “3-CAT kit Research ELISA” (LDN Labor Diagnostika, Nordhorn, Germany). This kit is a competitive immunoassay. The absorbance was read at 450 nm and the results were expressed in μg A/10^6^ peritoneal leukocytes, μg NA/10^6^ peritoneal leukocytes, and μg DA/10^6^ peritoneal leukocytes. A = adrenaline, NA = noradrenaline, DA = dopamine.

### 4.9. Statistical Analysis

The statistical analysis was performed in GraphPad Prism 8.4.1. (LLC, San Diego, CA, USA). Data were represented as mean ± standard deviation (SD). The normality of the samples and homogeneity of the variances were checked using the Kolmogorov–Smirnov test and Levene test, respectively. Comparisons between the groups were made by the independent-samples t-test according to the compatibility of the data with a normal distribution, and comparisons between results after the stress condition were made by the dependent-samples *t*-test, and *p* ≤ 0.05 was considered statistically significant.

## Figures and Tables

**Table 1:** Sensorimotor abilities and anxiety-like behaviors evaluated in female and male WT and TH-HZ mice in basal and post-stress conditions.

	Females				Males			
	WT Basal	WT Post-Stress	TH-HZ Basal	TH-HZ Post-Stress	WT Basal	WT Post-Stress	TH-HZ Basal	TH-HZ Post-Stress
Weight (g)	39 ± 2	39 ± 1	38 ± 3	40 ± 2	41 ± 1	40 ± 1	40 ± 2	42 ± 1
Reflex Visual placing reflex % Mice showing this response Hindlimb extensor reflex % Mice showing this response	100 100	100 100	100 100	100 100	100 100	100 100	100 100	100 100
Wood rod test Motor coordination Latency to leave the starting segment (s) Total number of crossing segments Time of permanence (s)	4 ± 2 3 ± 1 16 ± 5	8 ± 5 5 ± 2 43 ± 18 *	10 ± 6 5 ± 2 23 ± 6	9 ± 11 2 ± 2 * a 48 ± 22 *	4 ± 1 3 ± 1 11 ± 3	5 ± 3 6 ± 2 * 35 ± 22 *	14 ± 4 aa 3 ± 1 28 ± 5 aaa	4 ± 2 *** 4 ± 3 46 ± 17 *
Equilibrium % Mice falling off the wood rod Latency to fall (s) % Mice that complete the test Time to complete the test (s)	0 0 100 (Figure 1A)	0 0 63	0 0 75	0 0 25 ** a	0 0 100	0 0 63	0 0 50 a	0 0 50
Other behaviors Number of freezings Time of freezing (s) % Mice performing freezing	0 0 0	1 ± 1 3 ± 1 ** 25	0 0 0	2 ± 1 3 ± 1 ** 63 ** a	0 0 0	0 0 0	0 0 0	1 ± 1 3 ± 1 * a 12 #
Tightrope test Motor coordination Total number of crossing segments Time of permanence (s)	3 ± 1 20 ± 4	2 ± 1 42 ± 18 *	3 ± 1 50 ± 8 aaa	1 ± 1 ** 31 ± 24	4 ± 1 24 ± 5	3 ± 1 28 ± 14	4 ± 1 50 ± 7 aaa	3 ± 1 ## 40 ± 16
Muscular vigor % Mice falling off the tightrope Latency to fall (s) % Mice that complete the test Time to complete the test (s)	12 29 ± 4 (Figure 1B) 21 ± 5	38 37 ± 5 * 10 ± 7 *	20 17 ± 2 aaa 45 ± 12 aa	63 * 22 ± 19 0 *** a	0 0 ### 23 ± 5	13 26 ± 6 *** ## 24 ± 7 ##	50 a 28 ± 4 aaa ### 0 aaa ###	36 23 ± 8 34 ± 14 ** ##
Traction (%) Low Medium Maximum	0 12 (Figure 1C)	13 0	0 30	50 * 13	0 0	0 13	20 60	0 # 25
Elevated plus maze Number of times mice explore open arms Number of times mice explore closed arms Time in open arms (s) Time in closed arms (s) Time in central platform (s) % Time in open arms % Time in closed arms % Time in central platform Number of rearings Time of rearings (s) Number of groomings Time of groomings (s)	7 ± 3 8 ± 2 (Figure 2A) (Figure 2B) 100 ± 16 16 ± 3 51 ± 12 33 ± 9 7 ± 3 12 ± 4 1 ± 1 4 ± 2	1 ± 1 ** 1 ± 1 *** 11 ± 16 *** 0.3 ± 1 *** 96 ± 4 *** 3.7 ± 1 *** 5 ± 2 7 ± 4 11 ± 2 *** 15 ± 3 ***	3 ± 2 a 17 ± 3 aaa 23 ± 8 aaa 5 ± 1 aaa 88 ± 7 aaa 8 ± 3 aaa 7 ± 2 9 ± 3 15 ± 4 aaa 17 ± 5 aaa	1 ± 1 * 3 ± 2 *** 6 ± 2 ** 0.3 ± 1 *** 95 ± 2 * 4.7 ± 1 * 6 ± 2 9 ± 2 18 ± 3 aa 27 ± 4 ** aaa	3 ± 1 # 14 ± 2 ### 74 ± 13 # 5 ± 2 ### 70 ± 6 ## 25 ± 3 18 ± 3 ### 25 ± 4 ### 3 ± 2 7 ± 2 #	1 ± 1 ** 1 ± 1 *** 11 ± 16 *** 0.3 ± 1 ** 96 ± 4 *** 3.7 ± 2 *** 5 ± 2 *** 6 ± 3 *** 11 ± 3 *** 18 ± 5 **	1 ± 1 aa 13 ± 4 78 ± 23 ## 1 ± 1 aa ### 68 ± 4 ### 31 ± 3 aa ### 14 ± 5 # 16 ± 4 aa ## 5 ± 3 ### 9 ± 2 ##	1 ± 1 3 ± 2 *** 14 ± 12 *** 0.3 ± 1 95 ± 3 *** 4.7 ± 1 *** 6 ± 2 ** 8 ± 3 ** 18 ± 2 *** aa 25 ± 4 *** a
Burial behavior Standard condition Number of intact pieces Number of moved pieces Number of buried pieces	2 ± 1 (Figure 2C) 6 ± 3	10 ± 1 *** 2 ± 1 *	5 ± 1 aaa 8 ± 2	6 ± 1 aaa 6 ± 1 aaa	1 ± 1 9 ± 1	2 ± 1 ### 7 ± 1 ** ###	4 ± 1 aaa 6 ± 2 a	8 ± 1 *** aaa ## 2 ± 1 ** aaa ###
Bizonal condition Number of intact pieces Number of moved pieces Number of buried pieces	3 ± 1 6 ± 2 2 ± 2	4 ± 1 4 ± 1 3 ± 1	1 ± 2 6 ± 1 6 ± 1 aa	1 ± 1 aaa 7 ± 1 aaa 5 ± 1 aa	1 ± 1 ## 5 ± 2 5 ± 1 #	1 ± 1 ### 7 ± 1 ### 6 ± 1 ###	3 ± 2 4 ± 1 ## 3 ± 1 aa ###	3 ± 1 aa ## 5 ± 1 aa ## 3 ± 1 aaa ##

Each value represents the mean ± standard deviation of values corresponding to 6 animals. * *p* < 0.05, ** *p* < 0.01, *** *p* < 0.001 compared to basal condition, a *p* < 0.05, aa *p* < 0.01, aaa *p* < 0.001 compared to WT. # *p* < 0.05, ## *p* < 0.01, ### *p* < 0.001 compared to females.

**Table 2:** Exploratory behaviors evaluated in female and male WT and TH-HZ mice in basal and post-stress conditions.

	Females				Males			
	WT Basal	WT Post-Stress	TH-HZ Basal	TH-HZ Post-Stress	WT Basal	WT Post-Stress	TH-HZ Basal	TH-HZ Post-Stress
Holeboard test Non-goal-directed behavior Vertical exploration Number of wall rearings Time of wall rearings (s) Number of central rearings Time of central rearings (s) Horizontal exploration Inner locomotion Middle locomotion External locomotion Total locomotion Inner locomotion average Middle locomotion average External locomotion average Total locomotion average % Middle locomotion % External locomotion % Total locomotion Other behaviors Number of groomings Time of groomings (s) Number of freezings Time of freezings (s) Goal-directed behavior Number of head dippings Time of head dippings (s)	16 ± 4 19 ± 5 9 ± 4 8 ± 3 48 ± 7 139 ± 11 169 ± 12 Figure 3A 12 ± 2 12 ± 3 10 ± 2 12 ± 2 13 ± 3 39 ± 5 47 ± 5 0 0 0 0 Figure 3B 53 ± 9	10 ± 3 * 11 ± 4 * 2 ± 2 ** 4 ± 3 * 17 ± 12 *** 53 ± 24 *** 128 ± 28 * 4 ± 2 *** 4 ± 1 *** 7 ± 1 * 5 ± 1 *** 7 ± 6 22 ± 13 * 71 ± 18 *** 2 ± 1 ** 5 ± 2 ** 2 ± 1 ** 7 ± 3 ** 32 ± 6 **	38 ± 5 aaa 41 ± 5 aaa 2 ± 1 aa 4 ± 1 a 17 ± 4 aaa 63 ± 5 aaa 174 ± 18 4 ± 1 aaa 5 ± 1 aa 9 ± 1 7 ± 1 aaa 6 ± 2 aa 25 ± 3 aaa 69 ± 5 aaa 12 ± 3 aaa 17 ± 9 aa 4 ± 3 a 5 ± 2 aa 11 ± 3 aaa	0 *** aaa 0 *** aa 0 ** 0 *** a 7 ± 6 ** 54 ± 23 144 ± 14 ** 1 ± 1 *** a 4 ± 2 7 ± 1 ** 6 ± 1 5 ± 4 21 ± 11 73 ± 15 16 ± 3 * aaa 24 ± 3 aaa 5 ± 1 aaa 6 ± 2 3 ± 1 *** aaa	20 ± 4 22 ± 3 5 ± 2 7 ± 2 38 ± 6 # 118 ± 12 # 196 ± 22 # 10 ± 1 10 ± 1 10 ± 2 10 ± 1 11 ± 1 34 ± 3 56 ± 3 ## 0 0 0 0 49 ± 15	8 ± 2 *** 10 ± 5 *** 0 ** 0 *** # 13 ± 10 *** 79 ± 12 *** # 212 ± 32 ### 3 ± 1 *** 7 ± 1 *** ### 11 ± 1 ### 8 ± 1 ** ### 4 ± 2 *** 26 ± 3 *** 70 ± 3 *** 5 ± 2 ** # 9 ± 2 *** ## 4 ± 2 ** 9 ± 1 *** 16 ± 3 ** ###	32 ± 5 aa 32 ± 5 aa # 1 ± 1 aa 1 ± 1 aaa ### 21 ± 7 aa 73 ± 11 aaa 176 ± 19 5 ± 2 aaa 6 ± 1 aaa 9 ± 1 7 ± 1 aaa 8 ± 2 a 27 ± 1 aa 65 ± 3 aaa 8 ± 5 a 8 ± 5 a 3 ± 2 a 3 ± 2 a 11 ± 3 aa	0 *** aaa 0 *** aa 0 0 6 ± 4 ** 31 ± 8 *** aaa 133 ± 20 ** aaa 2 ± 1 * 3 ± 2 * aa 7 ± 1 ** aaa 5 ± 1 ** aaa 4 ± 2 ** 18 ± 3 *** aaa 78 ± 5 *** aa 10 ± 2 aa ## 16 ± 1 * aaa ### 8 ± 4 * 12 ± 3 *** ## 5 ± 1 ** aaa ##
T-Maze test Horizontal exploration Intersection time (s) Exploratory efficacy (s) Vertical exploration Number of rearings Time of rearings (s) Other behaviors Number of groomings Time of groomings (s) Number of freezings Time of freezings (s)	7 ± 1 Figure 3C 2 ± 1 3 ± 2 0 0 0 0	5 ± 2 1 ± 1 3 ± 1 1 ± 1 2 ± 1 ** 0 0	13 ± 1 aaa 2 ± 1 2 ± 1 0 0 0 0	10 ± 3 aa 0 ** 0 ** aaa 3 ± 1 *** aa 8 ± 2 *** aaa 2 ± 1 ** aa 5 ± 2 ** aa	4 ± 2 # 9 ± 2 ### 10 ± 2 ### 0 0 0 0	6 ± 1 1 ± 1 *** 3 ± 1 *** 1 ± 1 2 ± 1 ** 0 0	21 ± 3 aaa ### 2 ± 1 aaa 3 ± 1 aaa 3 ± 5 8 ± 2 aaa ### 2 ± 1 aa ## 6 ± 1 aaa ###	8 ± 2 *** 0 ** 0 *** aaa 1 ± 1 ## 10 ± 2 aaa 5 ± 1 *** aaa ### 7 ± 2 aaa
Corner test Number of corners Number of wall rearings Number of groomings Number of scratches	Figure 3D 2 ± 1 1 ± 1 3 ± 2	5 ± 1 *** 1 ± 1 2 ± 1	7 ± 3 aa 1 ± 1 a 0	4 ± 2 1 ± 1 1 ± 1	5 ± 2 # 1 ± 1 2 ± 1	4 ± 1 3 ± 2 1 ± 1	6 ± 1 1 ± 1 2 ± 1 ##	3 ± 2 * 10 ± 2 *** aaa ### 1 ± 1

Each value represents the mean ± standard deviation of values corresponding to 6 animals. * *p* < 0.05, ** *p* < 0.01, *** *p* < 0.001 compared to basal condition, a *p* < 0.05, aa *p* < 0.01, aaa *p* < 0.001 compared to WT. # *p* < 0.05, ## *p* < 0.01, ### *p* < 0.001 compared to females.

**Table 3:** Immune functions evaluated in peritoneal leukocytes of female and male WT and TH-HZ mice in basal and post-stress conditions.

	Females				Males			
	WT Basal	WT Post-Stress	TH-HZ Basal	TH-HZ Post-Stress	WT Basal	WT Post-Stress	TH-HZ Basal	TH-HZ Post-Stress
Macrophage functions Chemotaxis index (C.I) Phagocytic efficacy (%) Phagocytic index	Figure 4A 74 ± 1 Figure 4B	85 ± 6 **	49 ± 4 aaa	54 ± 11 aaa	75 ± 3	68 ± 6 * ###	57 ± 3 aaa ##	48 ± 4 ** aaa
Lymphocyte functions Chemotaxis index (C.I) Lymphoproliferation Basal response (c.p.m) LPS response (c.p.m) ConA response (c.p.m)	1119 ± 124 Figure 4D 4336 ± 135 Figure 4C	739 ± 128 *** 4235 ± 284	758 ± 67 aaa 3249 ± 294 aaa	197 ± 54 *** aaa 1254 ± 289 *** aaa	654 ± 76 ### 3897 ± 281 #	586 ± 17 # 3546 ± 322 ##	299 ± 36 aaa ### 2199 ± 255 aaa ###	256 ± 22 * aaa # 1239 ± 233 *** aaa
Natural Killer activity (%)	42 ± 2	67 ± 3 ***	28 ± 3 aaa	31 ± 4 aaa	35 ± 3 ##	37 ± 6 ###	26 ± 2 aaa	17 ± 4 ** aaa ###
Antioxidant compounds Catalase activity (UI CAT/10^6^ cells) Glutathione reductase activity (mU GR/10^6^ cells) Glutathione peroxidase activity (mU GPx/10^6^ cells) Reduced glutathione levels (nmol GSH/10^6^ cells)	19 ± 3 63 ± 12 Figure 5A Figure 5B	16 ± 3 367 ± 28 ***	10 ± 3 aaa 39 ± 15 a	7 ± 3 aaa 57 ± 12 * aaa	14 ± 3 # 47 ± 10 #	21 ± 2 ** ## 59 ± 5 * ###	9 ± 6 27 ± 11 aa	9 ± 4 aaa 74 ± 17 ***
Oxidant compounds Xanthine oxidase activity (U XAO/10^6^ cells) Oxidized glutathione levels (nmol GSSG/10^6^ cells)	Figure 5C 1.25 ± 0.39	2.47 ± 0.12 ***	2.55 ± 0.98 a	4.46 ± 0.23 ** aaa	1.88 ± 0.23 ##	3.45 ± 0.23 *** ###	2.82 ± 0.13 aaa	4.54 ± 0.13 *** aaa
Redox state indicator GSSG/GSH Ratio	Figure 5D							

Each value represents the mean ± standard deviation of values corresponding to 6 animals. * *p* < 0.05, ** *p* < 0.01, *** *p* < 0.001 compared to basal condition, a *p* < 0.05, aa *p* < 0.01, aaa *p* < 0.001 compared to WT. # *p* < 0.05, ## *p* < 0.01, ### *p* < 0.001 compared to females.

## Data Availability

Data will be send under request.
